# Comparison between the six-minute walk test and the six-minute step test in post stroke patients

**DOI:** 10.1186/1755-7682-6-31

**Published:** 2013-08-07

**Authors:** Talita Dias da Silva, Rodrigo Daminello Raimundo, Celso Ferreira, Camila Torriani-Pasin, Carlos Bandeira de Mello Monteiro, Osmar Aparecido Theodoro Júnior, Vitor E Valenti, Fernando Adami, Eliane Pires de Oliveira, Viviani Barnabé, Luiz Carlos de Abreu

**Affiliations:** 1 Universidade Federal de São Paulo, UNIFESP, R. Botucatu, SP 740, 03828-000, Brazil; 2 Escola de Educação Física e Esporte da Universidade de São Paulo, EEFE/USP, Av. Prof. Mello de Morais, 65, 05508-030 SP, Brasil; 3 Escola de Artes, Ciências e Humanidades da Universidade de São Paulo EACH/USP, Rua Arlindo Béttio, SP 1000,03828-00 SP, Brasil; 4 Faculdades Metropolitanas Unidas, FMU, Rua Taguá 150 - Prédio 1, 01508-010 SP, Brasil; 5 Programa de Pós-Graduação em Fisioterapia, Faculdade de Ciências e Tecnologia, Universidade Estadual Paulista, UNESP, Rua Roberto Simonsen, 305,19060-900 Presidente Prudente, SP, Brasil; 6 Departamento de Morfologia e Fisiologia, Faculdade de Medicina do ABC, Av. Príncipe de Gales, 821 09060-650 Santo André, SP, Brasil; 7 Departamento de Saúde da Coletividade, Faculdade de Medicina do ABC, Av. Príncipe de Gales, 821, Santo André, SP, Brasil; 8 Faculdade Mario Schenberg, Estrada do Espigão, SP, 06710540 Cotia, Brasil

**Keywords:** Exercise test, Mobility limitation, Stroke

## Abstract

**Background:**

The Stroke remains one of the major chronic diseases worldwide, and is considered a major cause of disability, which results not only in persistent neurological deficits, but also in the high physical deconditioning, nevertheless there are not many forms of assessing functional capacity in this population. We aimed to investigate the feasibility of the Six Minute Walk Teste and the Six-Minute Step Test (6MST) in post-stroke patients and compare the behavior of physiological variables during the 6MST and the Six-Minute Walk Test (6MWT), by correlating the functional performance obtained in both tests.

**Method:**

The 6MWT was carried out according to the American Thoracic Society (ATS) and the 6MST was performed in six minutes in order to compare it to the 6MWT in a 20 cm step. Was included post-stroke individuals able to walk without aid. All of them did the 6MWT and the 6MST.

**Results:**

12 patients participated in the study. There was no statistical difference in the parameters analyzed when tests were compared. There was poor correlation between the functional performance in both tests.

**Conclusion:**

The 6MWT and the 6MST is feasible for post-stroke patients and physiological responses are equal during the performance of both tests. However, there was no correlation with respect to functional performance, which was assessed by the distance walked in the 6MWT and by the number of steps climbed in the 6MST.

## Background

Stroke still is one of the major chronic diseases around the world [1-4]. It is also considered one of the main causes of disabilities [5] resulting not only in persistent neurological deficits, but also in a high physical deconditioning that propagates disability, leading to metabolic abnormalities that significantly increase the risk of myocardial infarction and recurrent stroke [6].

Among other physical disabilities, the functional walk is compromised after the stroke [7]. A factor that functionally compromises walking after stroke is the decrease in resistance, that is, the lack of aerobic conditioning [8]. This level of deconditioning, in combination with an increase in the demand of energy when walking, probably limits the locomotor performance in daily life activities [9], therefore affecting their independence and participation in community [10]. Although most post-stroke patients recover the ability to walk over the months following the vascular event, they are not physically able to walk socially, which affects their functional capacity [11]. Nevertheless, it is difficult to assess through objective and functional measures the impact of stroke on functional capacity and its consequences in the performance of daily activities.

Among some measures frequently used to determine the functional capacity [12,13], are the functional tests such as the Six-Minute Walk Test (6MWT) [14] and the Six-Minute Step Test (6MST). The use of these tests as determinants of the functional capacity may provide physiological parameters to determine the intensity of the exercises proposed during the rehabilitation of post-stroke patients.

The 6MST differs from the 6MWT, because allows the exercise capacity to be determined during routine consultations, increasing the frequency of functional assessment of patients, facilitating its application for it does not require much physical space [15].

Considering the above, the purpose of this study is to investigate the feasibility of conducting the 6MST and the 6MWT in post-stroke patients and compare the behavior of the physiological variables during the 6MST and the 6MWT.

## Method

This study evaluated 12 individuals with diagnosis of stroke who were being assisted by the physiotherapy services of the clinical school of the University. Walking without aid was established as the criterion for inclusion. The study was approved by the ethics committee with research protocol no. 13562439, CAAE, 0387.0.000.186-11. All patients signed an informed consent form before performing the tests. There were no sample losses.

In order to characterize the sample, scales Fugl-Meyer (FM) [16], Orpington [17], and Mini Mental State Examination (MMSE) [18] were applied in addition to the collection of personal data about age, gender, type, and side of injury.

The 6MWT was carried out according to the American Thoracic Society –ATS standards [19]. Additionally, the calculation of the Double Product was made. The 6MST was performed in six minutes in order to be compared to the 6MWT, the step was 20 cm high, 90 cm wide, and 30 cm long. Patients climbed up and down and all of them used support for the upper limbs, measuring the same physiological variables of the 6MWT.

We decided not to do both tests on the same day in order to prevent differences in perceived exertion and vital signs. Patients were interspersed according to the test that would be performed first.

### Statistical analysis

Statistical Analysis were performed by the SigmaStat software version 3.5 and the Kolmogorov-Smirnov was applied to the quantitative variables in order to verify if the distribution of variables was normal. The Levine's test was used to verify the homogeneity of variances. The paired t-test and/or t-test was used for variables Oxygen Saturation, Diastolic Blood Pressure, Heart Frequency, Respiratory Frequency, Borg Scale for dyspnea and lower limb fatigue, and Double Product, which presented normal distribution. For Systolic Blood Pressure, Difference of Systolic Blood Pressure, and Difference of Double Product variables, Wilcoxon test and/or Mann–Whitney test was used. Spearman coefficient was used to correlate the distance walked and the number of steps. To evaluate the correlation between physiological and hemodynamic measures on the 6MWT and 6MST, we used the intraclass correlation coefficient (ICC), Pearson correlation coefficient and Bland and Altman approach. A significance level of 0.05 (5%) with confidence intervals of 95% was set for this study.

## Results

As to the descriptive analysis, the 12 patients (6 men and 6 women) had a mean age of 54 ± 9 years. The mean age for the male gender was 54 years and for female gender was 55.5 years. The mean weight was 74 kilograms with average height of 1.66 meter.

Characterization data are shown in Table 1.

**Table 1:** Sample characterization

**Subjects**	**Gender**	**Age**	**Side of injury**	**Type of injury**	**Time of injury (years)**	**Total Fugl Meyer**	**MMSE**	**Orpington**		**DW 6MWT**	**Steps ST**
								**Score**	**Classification**		
**1**	M	40	R	I	5	152	29	2.0	Mi	303	69.5
**2**	F	42	R	I	2	176	25	1.7	Mi	171	78
**3**	F	64	L	H	36	142	30	3.3	Mo	363	53
**4**	M	52	L	I	4	146	5	5.4	S	222	27.5
**5**	F	54	R	H	8	201	20	3	Mi	279	47.5
**6**	M	66	R	I	7	152	20	4	Mo	225	63
**7**	F	64	L	I	6	173	26	2.6	Mi	332	116
**8**	M	61	L	I	6	163	29	3.6	Mo	270	55
**9**	M	56	L	I	4	173	20	3	Mi	253	67
**10**	M	43	L	I	3	154	28	2.2	Mi	256	76
**11**	F	54	R	H	4	174	26	1.8	Mi	330	79
**12**	F	57	L	I	8	154	6	3.8	Mo	286	59

*M* male gender, *F* female gender, *D* brain injury on the right hemisphere, *L* brain injury on the left hemisphere, *I* ischemic stroke, *H* hemorrhagic stroke, *MMSE* Mini Mental State Examination, *ROM* range of motion, *Mi* mild, *Mo* moderate, *S* severe, *DW6MWT* distance walked in six-minute walk test), *ST* step test.

As to the results of the feasibility of conducting the 6MST and the 6MWT in post-stroke individuals, all of them understood the instructions and concluded the first test proposed properly.

In the comparison of the physiological variables (beginning and end of the test) for the 6MWT there was a statistically significant difference for all variables, except for Diastolic Blood Pressure and Oxygen Saturation (Table 2).

**Table 2:** Description of the physiological variables in the 6MWT and the 6MST

	**6MWT**			**6MST**			**Difference final-initial between 6MWT × 6MST**		
	**Beginning**	**End**	***p***	**Beginning**	**End**	***p***	**ICC**	***r***	**Limits of agreement (lower. upper )**^**a**^
**SaO**_**2 **_**(%)**	96 ± 1.5	97 ± 1.5	0.046	97	97	0.938	0.262	0.335	−1.847 ; 3.014
**SBP (mmHg)**	115*	130*	0.039	120*	135*	<0.001	0.458	0.673	- 39.093 ; 14.093
**DBP, (mmHg)**	75 ± 5	75 ± 5	0.851	74 ± 6	80 ± 6	0.025	0.471	0.589	−30.759 ; 20.759
**HR, (bpm)**	77 ± 2	95 ± 2	<0.001	77 ± 9	97 ± 15	0.001	0.376	0.449	−30.984 ; 26.151
**RR, (rpm)**	17 ± 2	23 ± 3	<0.001	20*	26*	<0.001	0.042	0.040	−8.999 ; 6.666
**BS (dyspnea)**	9*	12*	0.003	8*	12*	<0.001	0.432	0.449	−6.942 ; 6.942
**BS (MMII)**	9*	14*	0.004	9*	13*	0.001	0.307	0.276	−9.283 ; 9.783
**DP (bat.mmHg.min**^**-1**^**)**	9295 ± 1868	12440 ± 3393	<0.001	9300*	11040*	<0.001	0.607	0.833	−7227.037 ; 3682.036

*6MWT* Six-Minute Walk Test, *6MST* Six-Minute Step Test, *SBP* Systolic Blood Pressure, *DBP* Diastolic Blood Pressure; HR: Heart Rate, *DP* Double Product, *RR* Respiratory Rate, *BS* Borg Scale, *SaO*_*2*_ Oxygen Saturation, *mmHg* millimeters of mercury, *bpm* beats per minute; *min* minute, *rpm* repetitions per minute, *LL* lower limbs, *ICC* Intraclass correlation coefficient, *r*: Pearson correlation coefficient; ^a^ Limits of agreement obtained by the Bland-Altman difference to Walk - Steps: (mean_dif_ -1.96* dp_dif_ and mean_dif_ +1.96*dp_dif_).

*Data in median.

Equally, there was statistically significant difference for all physiological variables in the comparisons from the beginning and end of the test for the 6MST (Table 2).

In order to compare both tests (6MWT × 6MST), we calculated the difference between the beginning and end through t-test or Mann–Whitney test. There was no statistical difference in the parameters analyzed when both tests were compared. Table 2 shows the behavior of physiological variables during the tests.

The Maximal Heart Rate (HRmax) and Submaximal Heart Rate (SHR) were calculated with HRmax: 220-age and the SHR: 85% of the HRmax and in both tests no patient reached HRmax.

As a complementary analysis, the following correlations were made: Lower Limbs, Balance and Total Fugl Meyer Scale × 6MWT and 6MST. In all correlations made, the Spearman coefficient was low, showing slight correlation (r < 0.3) between the variables (*p* > 0,05).

## Discussion

This study was carried out in order to investigate the feasibility of using the 6MST and 6MWT in post-stroke patients and compare the behavior of the physiological variables during both tests, by correlating the functional performance obtained.

We could observe that all patients assessed in this study completed the test properly, without pausing during the six minutes, without sudden drops in Oxygen Saturation, and without need of supplemental oxygen. Thus, the same way that the 6MST and the 6MWT proved to be reliable and reproducible in patients with pulmonary diseases [15], it is possible to say that it is a low cost method for assessing the current level of functional impairment and disability concerning primary care of the population of patients with stroke.

Averages for HRmax and SHR did not reach high values, reaching 56% and 66%, respectively, indicating that the 6MWT does not represent a maximal exercise test. These findings are confirmed in the study by Eng et al. [20] in which patients reached 63% of their HRmax on average. This information concerning the HR in the comparisons made at the end and beginning of the test are below the expected variations and may be explained to the extent post-stroke individuals present low cadence and, therefore, low average walking speed, which does not allow the establishment of the demand needed to increase Heart Rate that is close to the maximal or submaximal. In the 6MST, the Heart Rate obtained in the test, was 60% of their HRmax, which as well did not represent a maximal exercise test.

When comparing the results of the physiological variables of the individuals assessed in both tests, no differences statistically significant were found. The review of the literature showed no references illustrating the behavior of physiological variables of post-stroke individuals in the 6MST. However, when such data were observed in other populations, as in the study by Dal Corso et al. [15] in patients with pulmonary diseases, it is possible to observe that the 6MST causes few alterations to the physiological variables in comparison to other functional tests.

Based on the study concerning the physiological variables, the 6MST can be conducted in patients with stroke as an alternative for cases in which 6MWT was not possible due to the lack of large space, for example [20]. In this context, the use of such test for the purpose of measuring energy consumption in certain physical activities becomes a possible alternative.

For the distance walked, the result obtained in our study was 274 m ± 54 m. Pohl et al. [11] described that there is no standard distance and changes in pulse and blood pressure for patients with stroke as there is in healthy adults. However, they show that the distance walked by the patients assessed in their study, who had a stroke in less than 28 days, was 215.8 meters ± 91.6 on average. On the other hand, in a study carried out by Kosak and Smith [21], patients with stroke, also about 28 days after the vascular accident, had an average distance of 150 meters. The values closest to the ones found in our study were those of Eng et al. [20], who assessed chronic patients with stroke for more than one year and observed a walked distance of 268 meters.

Studies show that the performance in the 6MWT may be influenced by motivation, cardiovascular function, and respiratory function. It may also be influenced by the motor impairment of the affected lower limb and by the lack of balance [11,21,22]. However, these findings were not observed in this study, as there was no correlation between the functional performance in the tests and the variables of sample characterization.

There was no correlation between the distance walked in 6MWT and the number of steps climbed in the 6MST. Such findings can be explained by the fact that walking is an activity very used in daily life of the sample in this study, as patients are independent for walking, including walking without aid. On the other hand, the movement of going up and down stairs may not be such a common activity for these patients, who present balance deficit, measured by activities in Fugl-Meyer.

## Conclusion

The 6MWT and the 6MST are feasible for post-stroke patients and physiological responses are equal during the performance of both tests (6MST and 6MWT). However, there was no correlation with respect to functional performance, which was assessed by the distance walked in the 6MWT and by the number of steps climbed in the 6MST. This study showed that the 6MWT and the 6MST may be used to evaluation and prognosis in post-stroke patients.

## Competing interest

The authors declare that they have no competing interest.

## Author’s contribution

All authors participated in the acquisition of data and revision of the manuscript. TDS, CF, CTP, CBMM, OATJ, EPO and RDR conceived of the study, determined the design, performed the statistical analysis, interpreted the data and drafted the manuscript. VEV, LCA and FA determined the design and drafted the manuscript. All authors read and gave final approval for the version submitted for publication.
